# Supplementary material for: Single-atom alloy catalysts designed by first-principles calculations and artificial intelligence
Source: Nat Commun. 2021 Mar 23;12:1833. doi: 10.1038/s41467-021-22048-9 (PMC7988173; doi:10.1038/s41467-021-22048-9)
Supplement: Supplementary file 1 — Supplementary Information [file 41467_2021_22048_MOESM1_ESM.pdf]

Supplementary Information to the manuscript: Single-Atom Alloy Catalysts Designed by First-Principles Calculations and Artificial Intelligence

Zhong-Kang Han,[1#] Debalaya Sarker,[1#] Runhai Ouyang,[2#] Aliaksei Mazheika,[3] Yi Gao,[4]* Sergey V. Levchenko[1]**

[1] *Center for Energy Science and Technology, Skolkovo Institute of Science and Technology, Skolkovo Innovation Center, Moscow, 143026, Russia*
[2] *Materials Genome Institute, Shanghai University, 333 Nanchen Road, Shanghai, 200444, P.R. China*
[3] *Technische Universität Berlin, BasCat − UniCat BASF JointLab, Hardenbergstraße 36, 10623 Berlin, Germany*
[4] *Shanghai Advanced Research Institute, Chinese Academy of Sciences, Shanghai, 201210, P. R. China*
[#] *These authors contributed equally.*

Correspondence Email: S.Levchenko@skoltech.ru; gaoyi@zjlab.org.cn; alex.mazheika@gmail.com

Supplementary Methods

All *ab initio* calculations were performed with the revised Perdew-Burke-Ernzerhof (RPBE) functional[1] as implemented in the all-electron full-potential electronic-structure code FHI-aims[2] using density-functional theory (DFT) and numerical atom-centered basis functions. *Light* numerical settings are used which are tested to be converged compared to *tight* numerical settings, resulting in accuracy in total energy differences within 0.05 eV per supercell. The choice of functional is validated based on a comparison of calculated H_2 adsorption energies to the available experimental results[3] (Supplementary Table 1). Spin-polarization effects are tested and included where appropriate for the system with Fe, Co, and Ni atoms. Slabs of at least nine metal layers were considered with the two to four bottom layers fixed, based on the convergence of BE_H and SE (within 0.05 eV) with respect to the thickness and supercell size of the slab. The lattice vector along the direction parallel to the vacuum gap was 50 Å. All atoms in the systems except for the fixed bottom have been allowed to relax until the maximum remaining force fell below 10^{-2} eV/Å. The climbing-image nudged elastic band (CI-NEB) algorithm is employed to identify the transition state structures.[4] H atom is placed at different non-equivalent high-symmetry sites close to the guest atom (Supplementary Figure 1), and the BE_H for the most favorable site is included in the data set. The host metal surfaces considered in this work to construct the training data set are Cu(100), Cu(310), Zn(0001), Cr(110), Pd(111), Pd(211), Pt(111), Rh(111), Ru(0001), Cd(0001), Ag(100), Ag(110), Ti(0001), Nb(210), and Ta(210) with more than three hundred points for each considered properties. All the DFT calculated BE_H, SE, and E_b can be found in the file "Supplementary Data 1".

For constructing the Φ_1, Φ_2, and Φ_3 feature spaces we made use of the set of algebraic/functional operators given in eq. 1.

$$\hat{H}^{(m)} \equiv \{+, -, \cdot, /, \log, \exp, \exp-, ^{-1}, ^{2}, ^{3}, \sqrt{}, \sqrt[3]{}, |-|\}, \quad (1)$$

The superscript *m* indicates that when applying $\hat{H}^{(m)}$ to primary features φ_1 and φ_2 a dimensional analysis is performed, which ensures that only physically meaningful combinations are retained (e.g. only primary features with the same unit are added or subtracted). All primary features included in this study were obtained either from the literature (see Supplementary Table 2), or from DFT calculations (see Supplementary Table 3). The values of the primary features for the training data sets can be found in the file “Supplementary Data 1” and the values of the primary features for all the high-throughput screening SAAC candidates can be found in the file “Supplementary Data 2”.

The sparsifying ℓ_0 constraint is applied to a smaller feature subspace selected by a screening procedure (sure independence screening (SIS)), where the size of the subspace is equal to a user-defined SIS value times the dimension of the descriptor. The SIS value is not an ordinary hyperparameter and its optimization through a validation data set is not straightforward. Ideally, one would want to search the entire feature space for the optimal descriptor. However, this is not computationally tractable since the computational cost of the sparsifying ℓ_0 constraint grows exponentially with the size of the searched feature space. Instead, the SIS value should be chosen as large as computationally possible. The reasonable SIS values were chosen based on the convergence of the training error.

To confirm the reliability of the SISSO model optimization approaches, the data were initially divided into training and test sets. The Pd(211) and Pt(111) based systems are used as test set while all the other data were contained in the training set. As mentioned in the main text, 10-fold cross validation (CV10) method was used to determine the dimensionality of the descriptor. First, the best descriptors were selected by SISSO based on only the training data. The RMSR and CV10 errors on the training set for the descriptors of BE_H, E_b, and SE are displayed in Supplementary Figure 3a. Second, the predictive power of the SISSO selected descriptors was tested using the test set. We display in Supplementary Figure 3b the distribution of errors on the training set and test set for the descriptors of BE_H (green box), E_b (blue box), and SE (cyan box). To check the predictive power of SISSO selected descriptors on different types of surfaces, we divide the test set into two groups: one with only flat surface and the other one with only stepped surface. In the first group, we considered a new transition metal of Pt which are not contained in the training set (Cu, Zn, Cr, Pd, Rh, Ru, Cd, Ag, Ti, Nb, and Ta), while in the second group, we considered a new surface cut of fcc(211) which is also not included in the training set. The root-mean-square errors, RMSEs, (maximum absolute errors, MAEs) of the SISSO selected descriptors for the test flat surface set are found to be BE_H: 0.10 eV (0.43 eV), E_b: 0.12 eV (0.62 eV), and SE: 0.22 eV (0.78 eV), while for the test stepped surface set are found to be BE_H: 0.11 eV (0.54 eV), E_b: 0.14 eV (0.71 eV), and SE: 0.24 eV (0.87 eV). The moderate errors of the RMSEs for both test flat surface set and test stepped surface set showed that the transferability of the descriptors is good. The little larger RMSEs and MAEs for test stepped surface set compared to that for test flat surface set can be rationalized by the fact that in the training set, more flat surfaces are considered compared to stepped surfaces. We would expect the errors for the test set to further decrease when more data are included in the training set. Thus, after confirming the reliability of the of the SISSO model optimization approaches, finally we included all the data into the training set for the SISSO selection of the best descriptors. As mentioned in the main text, the obtained optimal descriptor dimensionalities for BE_H, E_b, and SE of the SAACs are 5, 6, and 6, respectively. The same optimal descriptor dimensionalities for BE_H, E_b, and SE were found when the Pt(111) and Pd(211) based

systems are not included, further confirming the reliability the used SISSO model optimization approaches. The error distributions for all the lower dimensional models are displayed in Supplementary Figures 4-6. The RMSE and MAE of the models are also shown. The identified lower-dimensional descriptors and the coefficients and correlations in corresponding SISSO models for BE_H, E_b, and SE are displayed in Supplementary Tables 4-6. The top five largest deviations between calculated and predicted BE_H, E_b, and SE are collected in Supplementary Table 7. As can be seen, the deviation for SE is larger than that of BE_H and E_b. However, we found even for SE_H our model's precision is higher than 95% (Supplementary Table 8).

Supplementary Table 1. The experimental and theoretical adsorption energies (in eV) of H_2 at different transition metal surfaces.

method	Pt(111)	Ru(0001)	Pd(111)
RPBE	-0.56	-1.06	-1.01
PBE	-0.32	-0.78	-0.70
Experiments[3]	-0.75	-1.20	-0.91

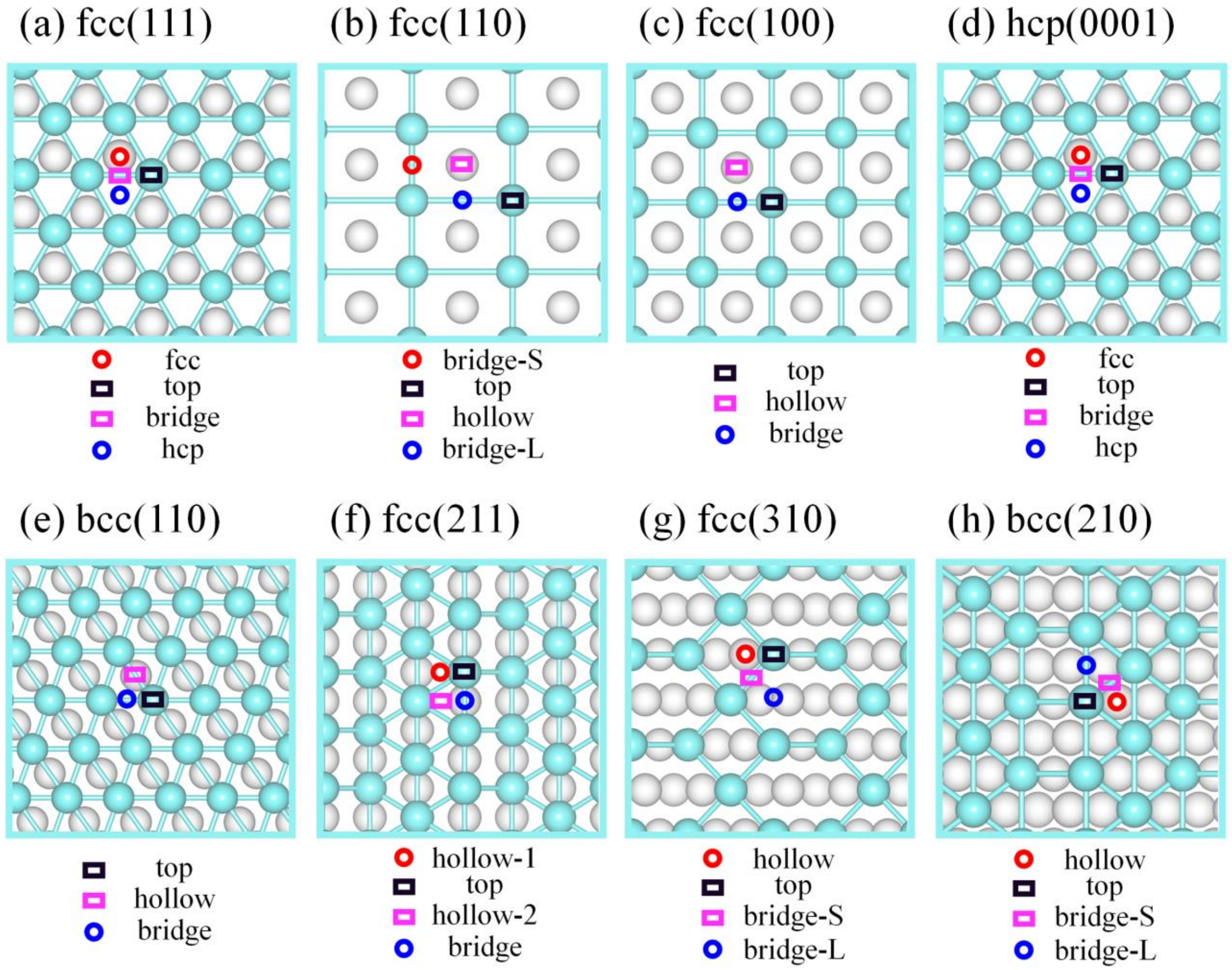

Supplementary Figure 1. The considered hydrogen adsorption sites on the fcc(111) (a), fcc(110) (b), fcc(100) (c), hcp(0001) (d), bcc(110) (e), fcc(211) (f), fcc(310) (g), bcc(210) (h) of pure transition metal surfaces. The atom below the top site for each surface facet is either the host atom (pure transition metal surfaces) or the single guest atom (single atom alloy metal surfaces).

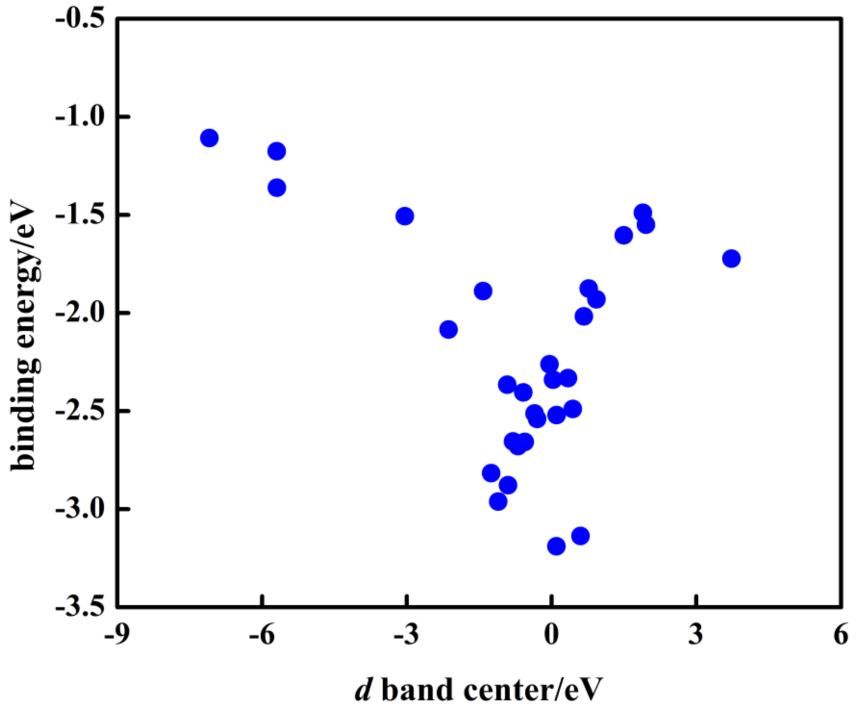

Supplementary Figure 2. Correlation between H-atom binding energy BE_H and the *d*-band center for Pt(111) based SAACs.

Supplementary Table 2. Primary features obtained from the literature.[5] Electron affinity (EA in eV), ionization potential (IP in eV), and covalent radius (R) of the metal atom.

system	class	name	abbreviation
host	atomic	Electron affinity	EA*
		Ionization potential	IP*
		Atomic radius	R*
guest atom	atomic	Electron affinity	EA
		Ionization potential	IP
		Atomic radius	R

Supplementary Table 3. Primary features obtained from DFT-RPBE calculations (spin-polarization effects are tested for and included where appropriate). Energy of the highest-occupied Kohn-Sham level (H in eV), energy of the lowest-unoccupied Kohn-Sham level (L in eV), binding energy of H with isolated metal atom (EH in eV as calculated by equation (2)), binding energy of

metal dimers (EB in eV as calculated by equation (3)), binding distance of H with isolated metal atom (dH in Å), and binding distance of metal dimers of the metal atom; cohesive energy (EC in eV as calculated by equation (4)) and *d*-band center (DC in eV) of the bulk metal; *d*-band center of the top surface layer (DT in eV), *d*-band center of the subsurface layer (DS in eV), and the slab Fermi level (F in eV) of the metal surface.

system	class	name	abbreviation
host	atomic	Energy of the highest-occupied Kohn-Sham level	H*
		Energy of the lowest-unoccupied Kohn-Sham level	L*
		Binding energy of H with single host metal atom	EH*
		Binding energy of host metal dimers	EB*
		Binding distance of H with single host metal atom	dH*
		Binding distance of host metal dimer	dd*
	bulk	Cohesive energy	EC*
		d-band center	DC*
	surface[#]	*d*-band center of the top surface layer	DT*
		d-band center of the subsurface layer	DS*
		Slab Fermi level	F*
guest atom	atomic	Energy of the highest-occupied Kohn-Sham level	H
		Energy of the lowest-unoccupied Kohn-Sham level	L
		Binding energy of H with single guest metal atom	EH
		Binding energy of guest metal dimers	EB
		Binding distance of H with single guest metal atom	dH
		Binding distance of guest metal dimers	dd
	bulk	Cohesive energy	EC
		d-band center	DC

[#]the surface based primary features were calculated by using the unit cell consisting of one atom per atomic layer.

$$EH = E_{\text{H-metal}} - E_{\text{metal}} - E_{\text{H}} \qquad (2)$$

where $E_{\text{H-metal}}$ is the energy of the total H-metal system, E_{metal} is the energy of the isolated metal atom, and E_{H} is the energy of the isolated H atom.

$$EB = E_{\text{dimer}} - 2E_{\text{single}} \qquad (3)$$

where E_{dimer} is the energy of the total metal-metal dimer system, E_{single} is the energy of the isolated metal atom.

$$EC = E_{\text{bulk}}/\text{n} - E_{\text{single}} \qquad (4)$$

where E_{bulk} is the energy of the bulk metal system, E_{single} is the energy of the isolated metal atom, and n is the number of metal atoms in the bulk unit cell.

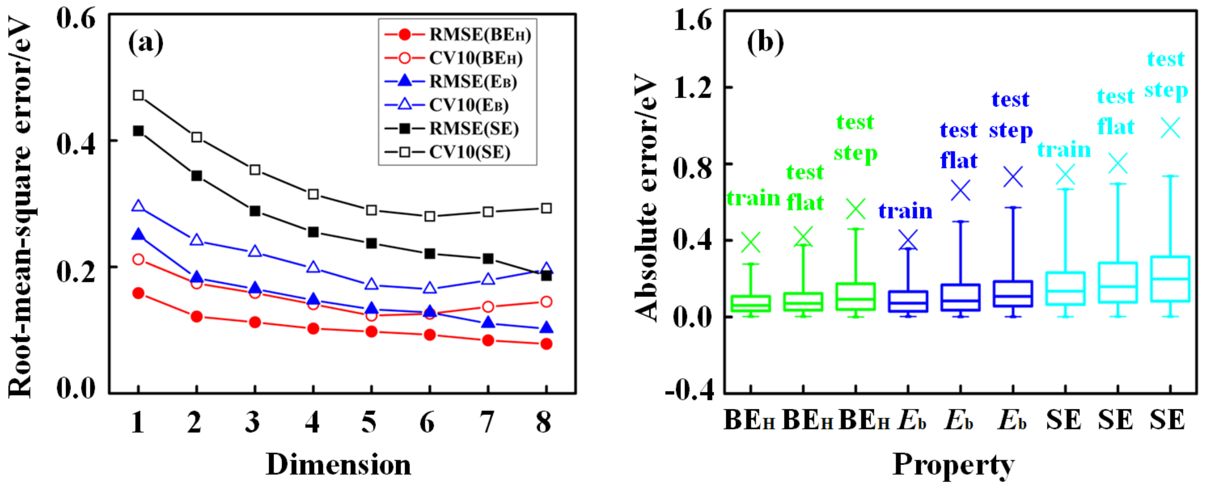

Supplementary Figure 3. (a) RMSE and the averaged RMSE of the 10 fold cross-validation. (b) Box plots of the absolute errors for the training set and test set for the SISSO selected best models of BE_H (green), E_b (blue), and SE (cyan). The test set is divided into two parts: one part contains only flat surfaces and the other one contains only stepped surfaces. The upper and lower limits of the rectangles represent the 75th and 25th percentiles of the distribution, the internal horizontal lines mark the median (50th percentile), and the upper and lower limits of the error bars indicate the 99th and 1st percentiles. The crosses depict the maximum absolute errors.

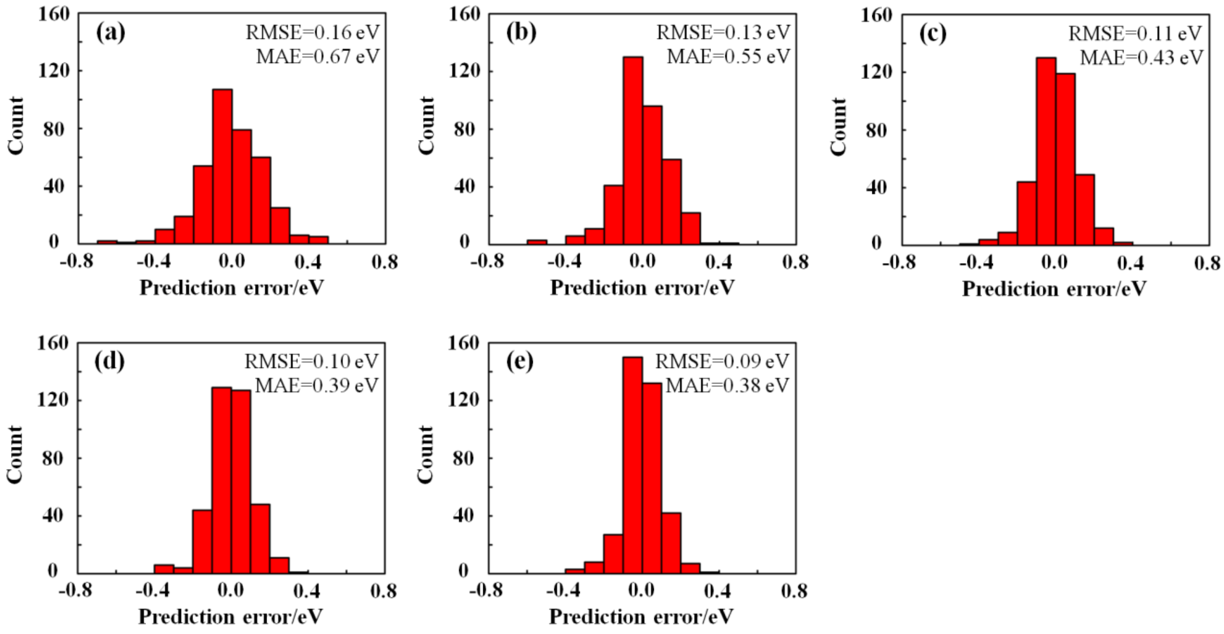

Supplementary Figure 4. The error distributions for all the lower-dimensional models of BE_H: (a) 1D, (b) 2D, (c) 3D, and (d) 4D, and the SISSO selected best model (e) 5D.

Supplementary Table 4. The identified descriptors and the coefficients and correlations for all the lower-dimensional models of BE_H.

d^m		descriptor	coefficient	correlation
d^1	d_1^1	(EA*+2F*−EC)·DT*·EH*/(EC*+F*)	0.12286E+00	0.8964
d^2	d_1^2	(EA*+2F*−EC)·DT*·EH*/(EC*+F*)	0.12093E+00	0.8964
	d_2^2	$\sqrt[3]{DC}$·H*·DT*·(|EA*−EH*|−|EC−EC*|)	-0.20756E-02	0.5891
d^3	d_1^3	(EA*+2F*−EC)·DT*·EH*/(EC*+F*)	0.12393E+00	0.8964
	d_2^3	$\sqrt[3]{DC}$·H*·DT*·(|EA*−EH*|−|EC−EC*|)	-0.19673E-02	0.5891
	d_3^3	|EH*−L*−|EH−F*||/(DC^2+EC·EC*)	-0.56460E+00	0.4850
d^4	d_1^4	(EA*+2F*−EC)·DT*·EH*/(EC*+F*)	0.11932E+00	0.8964
	d_2^4	$\sqrt[3]{DC}$·H*·DT*·(|EA*−EH*|−|EC−EC*|)	-0.18876E-02	0.5891
	d_3^4	|EH*−L*−|EH−F*||/(DC^2+EC·EC*)	-0.60955E+00	0.4850
	d_4^4	|IP*·F*/(EH+F*)−|EC*−DS*|−|DC*−F*||	0.31619E-01	0.3864
d^5	d_1^5	(EA*+2F*−EC)·DT*·EH*/(EC*+F*)	0.12653E+00	0.8964
	d_2^5	$\sqrt[3]{DC}$·H*·DT*·(|EA*−EH*|−|EC−EC*|)	-0.20440E−02	0.5891
	d_3^5	|EH*−L*−|EH−F*||/(DC^2+EC·EC*)	-0.50891E+00	0.4850
	d_4^5	|EH−F*−EH*|−|EC*−EC−|DT*−F*||	0.34705E−01	0.3849
	d_5^5	L·EC·(EA*+DS*−|H−EH|/|L*−EH*|	-0.48772E−04	0.3862

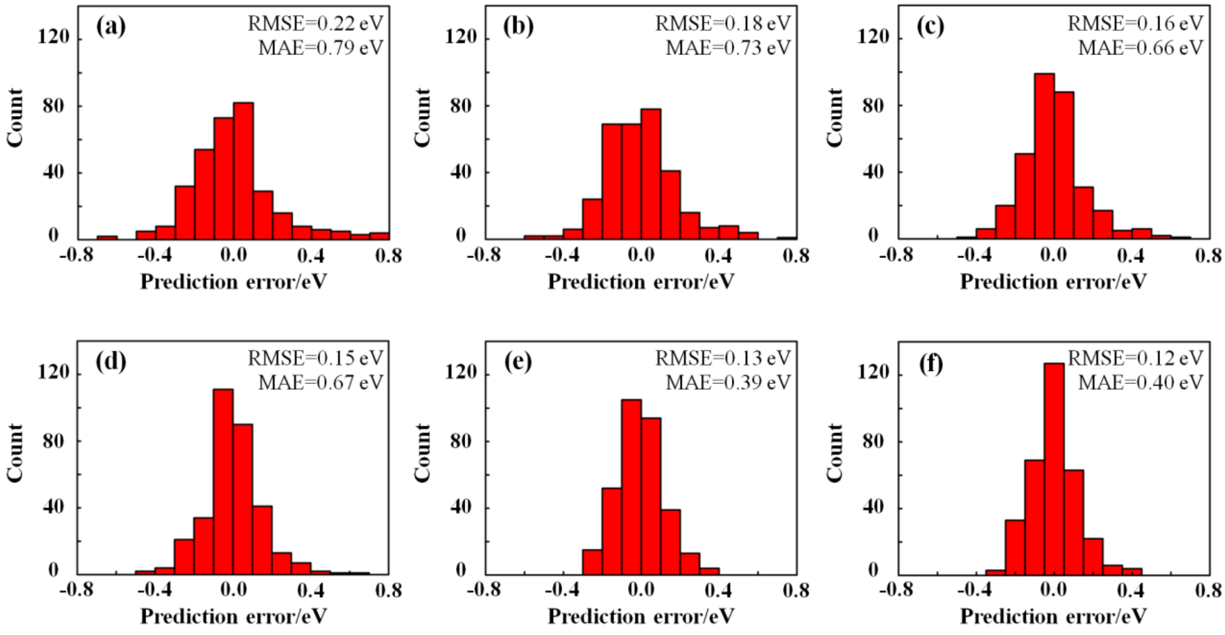

Supplementary Figure 5. The error distributions for all the lower dimensional models of E_b: (a) 1D, (b) 2D, (c) 3D, (d) 4D, (e) 5D and the SISSO selected best model (f) 6D.

Supplementary Table 5. The identified descriptors and the coefficients and correlations for all the lower-dimensional models of E_b.

d^m		descriptor	coefficient	correlation
d^1	d_1^1	((IP*−L)−|EC*−DT*|)/|EC/DC−L*/IP*|	0.92945E-01	0.7643
d^2	d_1^2	((IP*−L)−|EC*−DT*|)/|EC/DC−L*/IP*|	0.98762E-01	0.7643
	d_2^2	(EA*+DC*+|DC−DT*|)/(EA*+EH*+|L*−F*|)	-0.23925E-01	0.5726
d^3	d_1^3	((IP*−L)−|EC*−DT*|)/|EC/DC−L*/IP*|	0.91192E-01	0.7643
	d_2^3	(EA*+DC*+|DC−DT*|)/(EA*+EH*+|L*−F*|)	-0.22522E-01	0.5726
	d_3^3	(DC+EH*)·(EC*−F*)·(|L−EC|−|EC−EH|)	-0.14550E-01	0.4568
d^4	d_1^4	((IP*−L)−|EC*−DT*|)/|EC/DC−L*/IP*|	0.90281E-01	0.7643
	d_2^4	(EA*+DC*+|DC−DT*|)/(EA*+EH*+|L*−F*|)	-0.21259E-01	0.5726
	d_3^4	(DC+EH*)·(EC*−F*)·(|L−EC|−|EC−EH|)	-0.14089E-01	0.4568
	d_4^4	(DT*−EH)·DC·(H/EC+EA*/L*)/EC*	-0.18463E-01	0.4414
d^5	d_1^5	((IP*−L)−|EC*−DT*|)/|EC/DC−L*/IP*|	0.84777E-01	0.7643
	d_2^5	(EA*+DC*+|DC−DT*|)/(EA*+EH*+|L*−F*|)	-0.18289E-01	0.5726
	d_3^5	(DC+EH*)·(EC*−F*)·(|L−EC|−|EC−EH|)	-0.13888E-01	0.4568
	d_4^5	(DT*−EH)·DC·(H/EC+EA*/L*)/EC*	-0.19258E-01	0.4414
	d_5^5	DC^2·DT*/IP·(L*−DS*+|H*−EC*|)	-0.57167E-02	0.3975
d^6	d_1^6	((IP*−L)−|EC*−DT*|)/|EC/DC−L*/IP*|	-0.87339E−01	0.7643
	d_2^6	(EA*+DC*+|DC−DT*|)/(EA*+EH*+|L*−F*|)	-0.19577E−01	0.5726
	d_3^6	(DC+EH*)·(EC*−F*)·(|L−EC|−|EC−EH|)	-0.13173E−01	0.4568
	d_4^6	(DT*−EH)·DC·(H/EC+EA*/L*)/EC*	-0.19172E−01	0.4414
	d_5^6	e^{EC}·EH·DS*/((L*−DS*)+|H*−EC*|)	0.33549E−01	0.3768
	d_6^6	DC^2·(EC*−F*)/(DT*−F*−EA+EC)	-0.14362E−02	0.3643

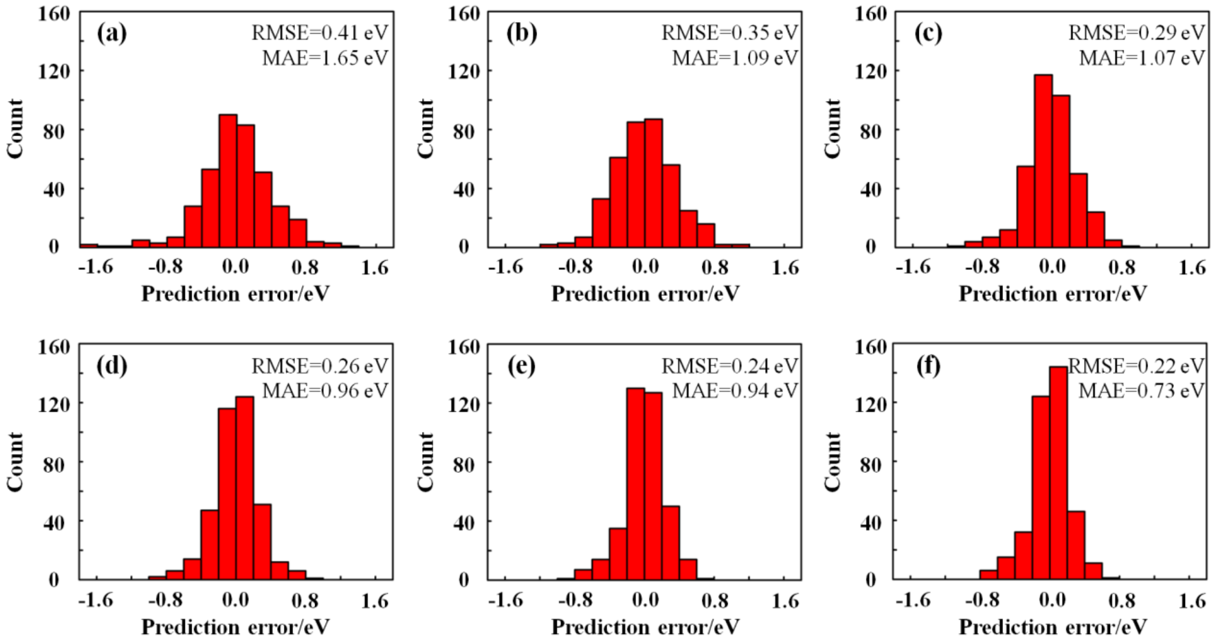

Supplementary Figure 6. The error distributions for all the lower dimensional models of SE: (a) 1D, (b) 2D, (c) 3D, (d) 4D, (e) 5D and the SISSO selected best model (f) 6D.

Supplementary Table 6. The identified descriptors and the coefficients and correlations for all the lower-dimensional models of SE.

d^m		descriptor	coefficient	correlation
d^1	d_1^1	(EC+IP+|F*−DT*|)/(IP*/R+H*/dd*)	-0.75479E+00	0.8969
d^2	d_1^2	(EC+IP+|F*−DT*|)/(IP*/R+H*/dd*)	-0.75372E+00	0.8969
	d_2^2	|DC−EB*|·(L−DC−EC)/EB^2	0.21861E+00	0.5346
d^3	d_1^3	(EC+IP+|F*−DT*|)/(IP*/R+H*/dd*)	-0.81257E+00	0.8969
	d_2^3	|DC−EB*|·(L−DC−EC)/EB^2	0.24095E+00	0.5346
	d_3^3	||EC*−L*|+|DC−DS*|−|DC−F*|−|EC−F*||	0.12052E+00	0.5386
d^4	d_1^4	(EC+IP+|F*−DT*|)/(IP*/R+H*/dd*)	-0.82171E+00	0.8969
	d_2^4	|DC−EB*|·(L−DC−EC)/EB^2	0.30402E+00	0.5346
	d_3^4	||EC*−L*|+|DC−DS*|−|DC−F*|−|EC−F*||	0.11671E+00	0.5386
	d_4^4	|H−IP−L+IP*|/((DC/EC)+(EC/H))	0.16261E+00	0.3913
d^5	d_1^5	(EC+IP+|F*−DT*|)/(IP*/R+H*/dd*)	-0.82332E+00	0.8969
	d_2^5	|DC−EB*|·(L−DC−EC)/EB^2	0.30382E+00	0.5346
	d_3^5	||EC*−L*|+|DC−DS*|−|DC−F*|−|EC−F*||	0.10926E+00	0.5386
	d_4^5	|H−IP−L+IP*|/((DC/EC)+(EC/H))	0.16324E+00	0.3913
	d_5^5	(F*−EC)·(L*−DT*−IP)/(F*−EB*)	-0.55996E-02	0.3982
d^6	d_1^6	(EC+IP+|F*−DT*|)/(IP*/R+H*/dd*)	-0.82665E+00	0.8969
	d_2^6	|DC−EB*|·(L−DC−EC)/EB^2	0.30742E+00	0.5346
	d_3^6	||EC*−L*|+|DC−DS*|−|DC−F*|−|EC−F*||	0.11317E+00	0.5386
	d_4^6	|H−IP−L+IP*|/((DC/EC)+(EC/H))	0.17455E+00	0.3913
	d_5^6	(F*−EC)·(L*−DT*−IP)/(F*−EB*)	-0.51761E−02	0.3982
	d_6^6	EC*·DC·(EB*−L)·(L+L*−EC−DS*)	-0.80032E−03	0.3379

Supplementary Table 7. The top five largest deviations between calculated and predicted BE_H (in eV), E_b (in eV), and SE (in eV).

property	system	calculated	predicted	deviation
BE_H	Co/Ag(100)	-2.98	-2.60	0.38
	Co/Ag(110)	-2.98	-2.65	0.33
	Ni/Ti(0001)	-2.95	-3.27	0.33
	Ta/Ti(0001)	-3.19	-2.89	0.30
	Sc/Zn(0001)	-1.91	-2.21	0.30
E_b	Hf/Pt(111)	0.78	0.38	0.40
	Cu/Ag(110)	0.92	0.52	0.40
	Ir/Zr(0001)	0.68	0.28	0.40
	Pd/Ag(110)	0.60	0.24	0.36
	Zr/Cu(100)	0.71	0.38	0.33
SE	V/Pd(111)	-0.27	0.46	0.73
	Hg/Zn(0001)	-0.24	0.48	0.71
	Os/Zn(0001)	0.96	0.25	0.71
	Cd/Zn(0001)	-0.13	0.50	0.62
	Hg/Cd(0001)	-0.19	0.43	0.62

Supplementary Table 8. Number of systems with the predicted and calculated SE_H that meet the same condition of $SE_H < kT\ln(10)$ (N_{meet}), the total number of calculated systems (N_{total}), and the SE model's precision ($P = N_{meet}/ N_{total}$).

temperature	N_{meet}	N_{total}	P
200 K	345	360	95.83%
700 K	346	360	96.11%

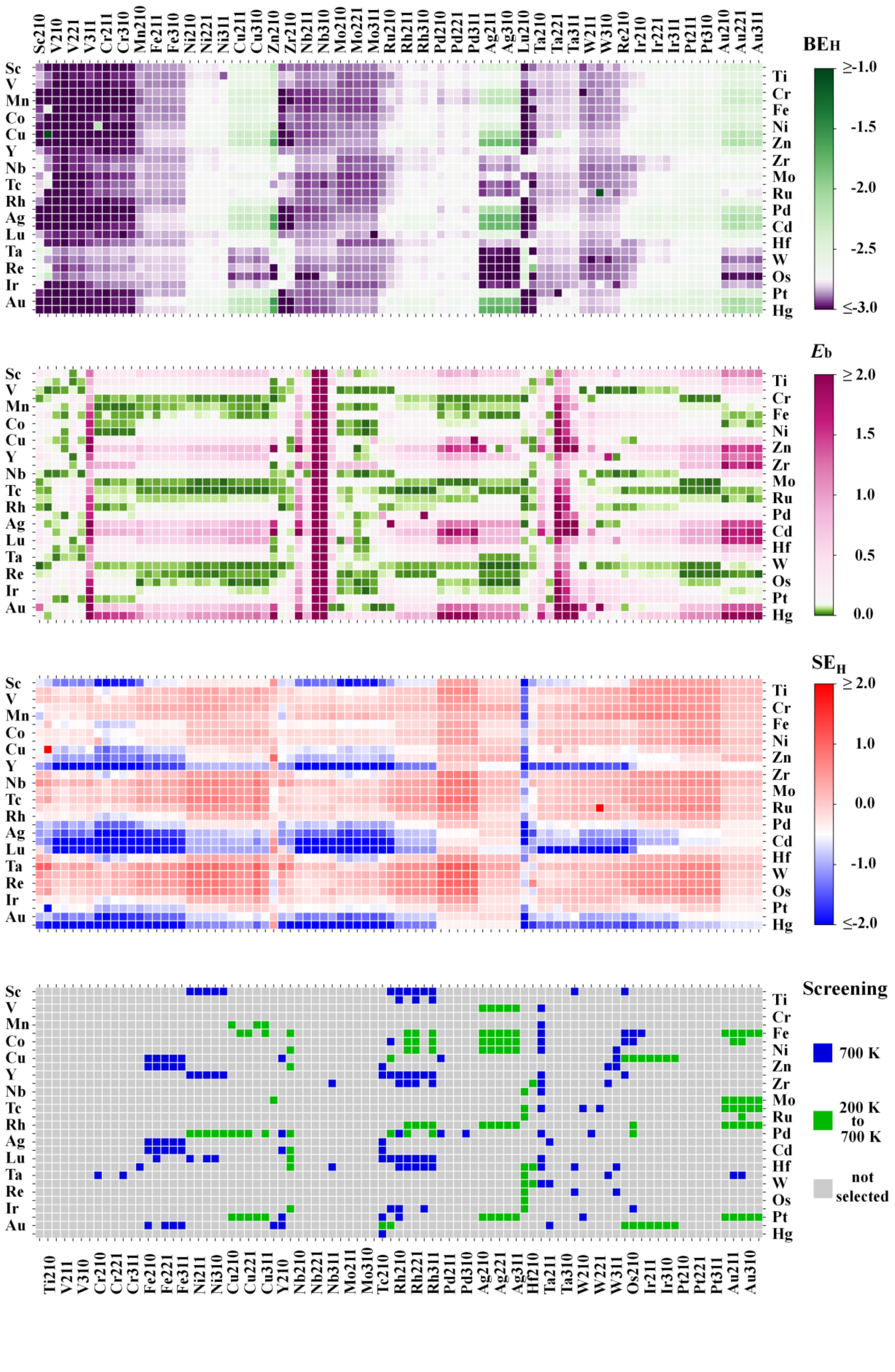
Sc210 V210 V221 V311 Cr211 Cr310 Mn210 Fe211 Fe310 Ni210 Ni221 Ni311 Cu211 Cu310 Zn210 Zr210 Nb211 Nb310 Mo210 Mo221 Mo311 Ru210 Rh211 Rh310 Pd210 Pd221 Pd311 Ag211 Ag310 Lu210 Ta210 Ta221 Ta311 W211 W310 Re210 Ir210 Ir221 Ir311 Pt211 Pt310 Au210 Au221 Au311
Sc V Mn Co Cu Y Nb Tc Rh Ag Lu Ta Re Ir Au
Ti Cr Fe Ni Zn Zr Mo Ru Pd Cd Hf W Os Pt Hg
BEH
≥-1.0
-1.5
-2.0
-2.5
≤-3.0
Eb
≥ 2.0
1.5
1.0
0.5
0.0
SEH
≥ 2.0
1.0
0.0
-1.0
≤-2.0
Screening
700 K
200 K to 700 K
not selected
Ti210 V211 V310 Cr210 Cr221 Cr311 Fe210 Fe221 Fe311 Ni211 Ni310 Cu210 Cu221 Cu311 Y210 Nb210 Nb221 Nb311 Mo211 Mo310 Tc210 Rh210 Rh221 Rh311 Pd211 Pd310 Ag210 Ag221 Ag311 Hf210 Ta211 Ta310 W210 W221 W311 Os210 Ir211 Ir310 Pt210 Pt221 Pt311 Au211 Au310

Supplementary Figure 7. High-throughput screening of SAACs for (a) BE_H, (b) E_b, and (c) SE_H. The screened candidates are highlighted in (d). Vertical axis displays the guest atom type, and the vertical horizontal axis displays the host metal surfaces with different stepped surface cuts.

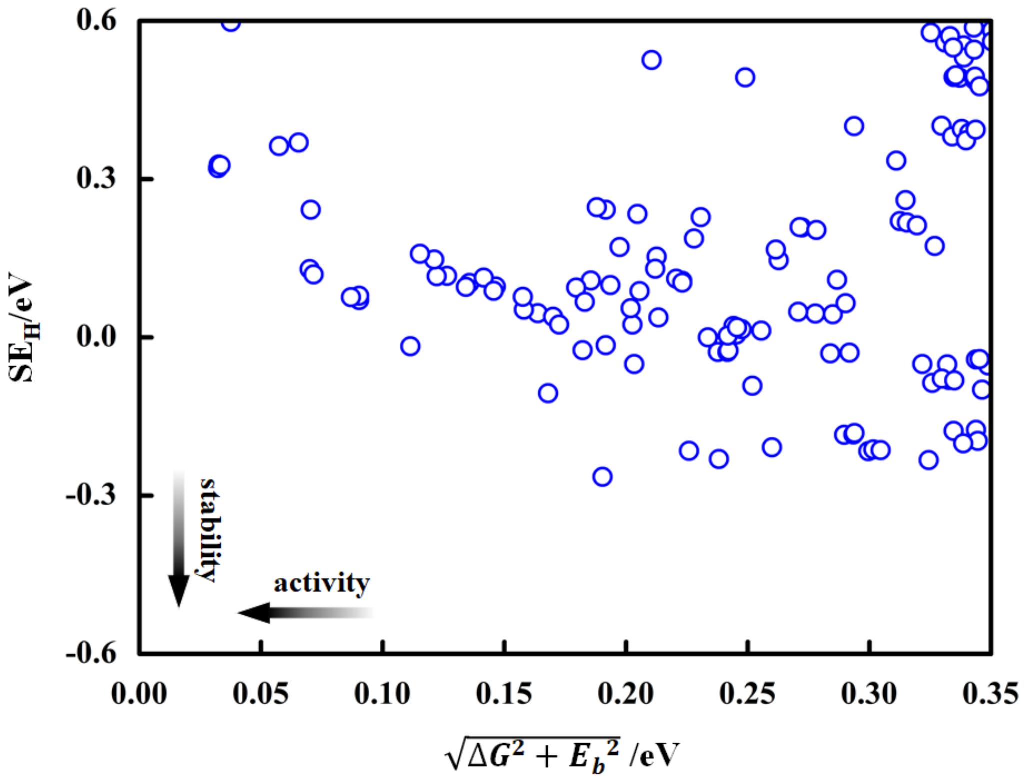

Supplementary Figure 8. Stability vs. activity map for stepped SAACs surfaces at $T = 298\ K$ and $p = 1$ atm. The SE_H on y-axis represents stability and activity parameter $\sqrt{\Delta G^2 + E_b^2}$ is shown on x-axis.

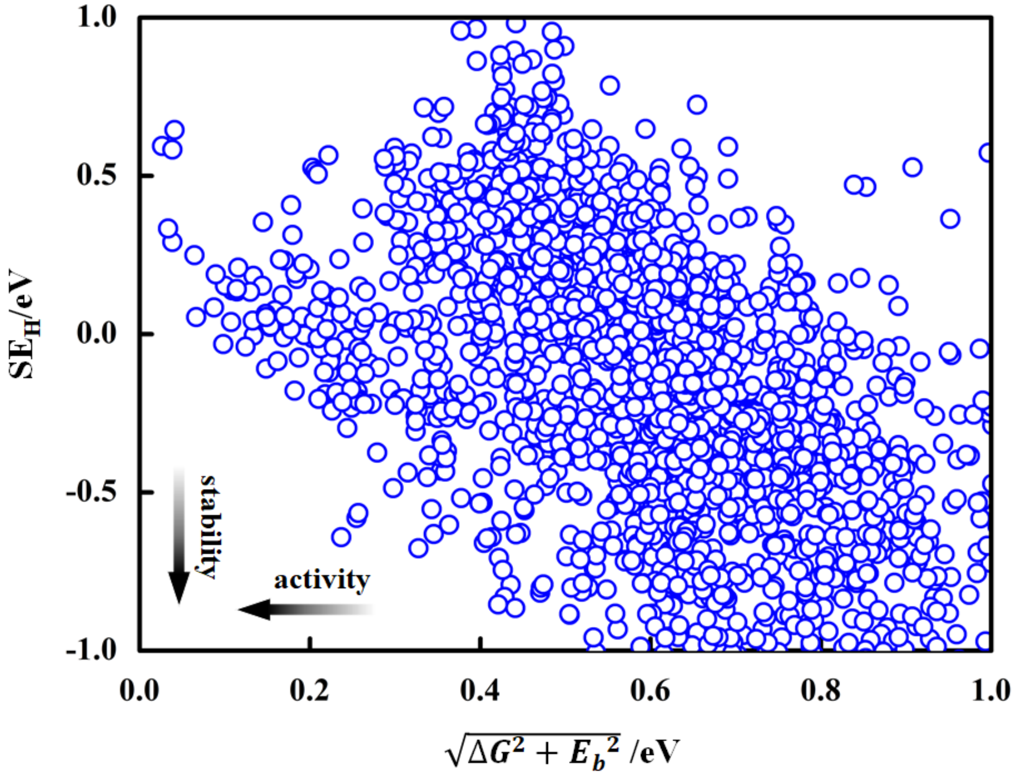

Supplementary Figure 9. Stability vs. activity map for SAACs surfaces at $T = 298\ K$ and $p = 1$ atm. The SE_H on y-axis represents stability and activity parameter $\sqrt{\Delta G^2 + E_b{}^2}$ is shown on x-axis.

Supplementary Table 9. The aggregation energies (E_A, in eV) for Mn/Ag(111), Pt/Zn(0001), and the experimentally established SAACs.

Systems	Mn/Ag(111)	Pt/Zn(0001)	Pd/Cu(111)	Pt/Cu(111)	Pd/Au(111)	Pt/Au(111)	Au/Ru(111)	Ni/(Zn(0001)
E_A	2.43	0.29	0.12	0.17	0.08	0.03	-0.16	0.16

The aggregation energies were calculated as the formation energy of guest atom dimer from isolated single guest atom. Positive values mean repulsive interactions between the two guest atoms.

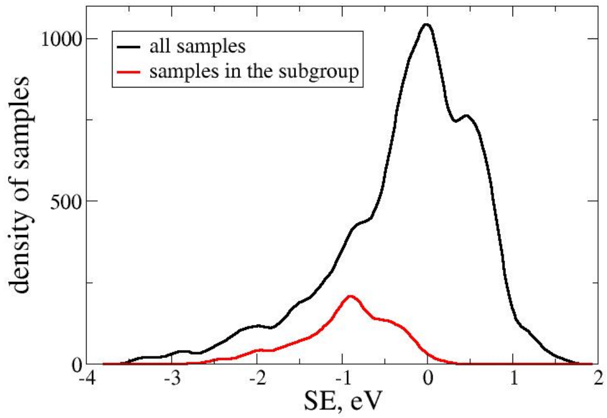

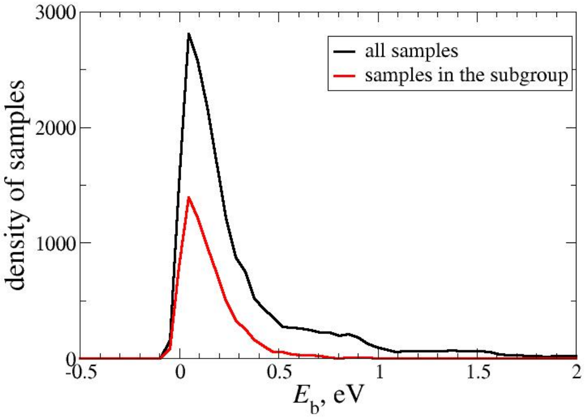

Supplementary Figure 10. The distribution of data samples for segregation energies (left) and H_2 dissociation energy barriers (right) in the whole sampling and obtained subgroups from SGD minimization of SE and $\sqrt{\Delta G^2 + E_b{}^2}$.

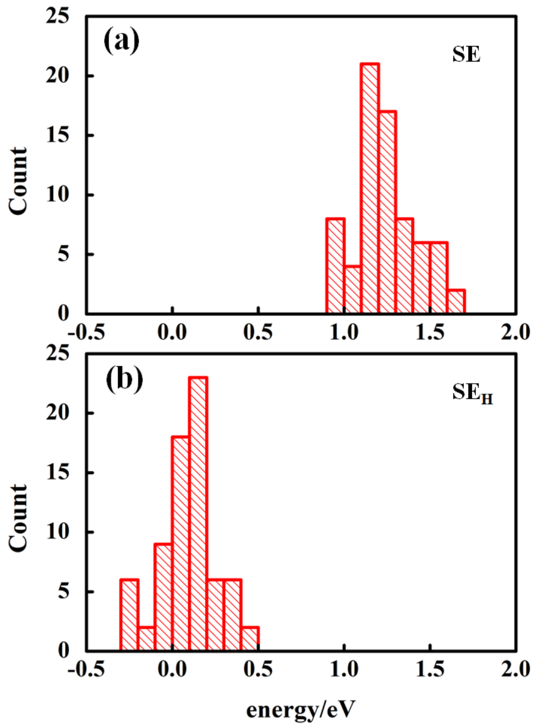

Supplementary Figure 11. The distribution of data samples for SE (a) and SE_H (b) for the subgroup (-2.35 eV ⩽ EH* ⩽ -2.32 eV) AND (EC* > -2.73 eV) AND (EC < -5.98 eV) AND (H ⩾ -5.12 eV) obtained from SGD minimization of SE_H – SE.

Supplementary References

1. Hammer, B., Hansen, L.B. & Nørskov, J.K. Improved adsorption energetics within density-functional theory using revised Perdew-Burke-Ernzerhof functionals. *Physical Review B* **59**, 7413 (1999).

2. Blum, V. et al. Ab initio molecular simulations with numeric atom-centered orbitals. *Computer Physics Communications* **180**, 2175-2196 (2009).
3. Silbaugh, T.L. & Campbell, C.T. Energies of formation reactions measured for adsorbates on late transition metal surfaces. *The Journal of Physical Chemistry C* **120**, 25161-25172 (2016).
4. Henkelman, G., Uberuaga, B.P. & Jónsson, H. A climbing image nudged elastic band method for finding saddle points and minimum energy paths. *The Journal of Chemical Physics* **113**, 9901-9904 (2000).
5. Dean, J.A. Lange's handbook of chemistry. (New york; London: McGraw-Hill, Inc., 1999).
