# Supplementary material for: Single-atom alloy catalysts designed by first-principles calculations and artificial intelligence
Source: Nat Commun. 2021 Mar 23;12:1833. doi: 10.1038/s41467-021-22048-9 (PMC7988173; doi:10.1038/s41467-021-22048-9)
Supplement: Supplementary file 3 — Description of Additional Supplementary Files [file 41467_2021_22048_MOESM3_ESM.pdf]

Description of Additional Supplementary Files

File Name: Supplementary Data 1
Description: Values of the primary features for the training data sets

File Name: Supplementary Data 2
Description: Values of the primary features for all the high-throughput screening SAAC candidates
